# Alzheimer’s disease: history, ethics and medical humanities in the context of assisted suicide

**DOI:** 10.1186/s13010-021-00111-z

**Published:** 2022-03-30

**Authors:** Birgit Braun, Joachim Demling, Thomas Horst Loew

**Affiliations:** 1 Department of Psychosomatic Medicine, University Hospital of Regensburg, Franz-Josef-Strauss-Allee 11, 93053 Regensburg, Germany; 2 Department of Psychiatry and Psychotherapy, University Hospital of Erlangen, Erlangen, Germany; 3 Department of Psychosomatic Medicine, University Hospital of Regensburg, Regensburg, Germany

**Keywords:** Alzheimer dementia, Dignity, Ethics, Assisted suicide, Integrative dementia therapy model

## Abstract

**Introduction:**

Dementia diseases, especially Alzheimer’s disease (AD), are of considerable importance in terms of social policy and health economics. Moreover, against the background of the current Karlsruhe judgement on the legalisation of assisted suicide, there are also questions to be asked about medical humanities in AD.

**Methodology:**

Relevant literature on complementary forms of therapy and prognosis was included and discussed.

**Results:**

Creative sociotherapeutic approaches (art, music, dance) and validating psychotherapeutic approaches show promise for suitability and efficiency in the treatment of dementia, but in some cases still need to be scientifically tested. Biomarker-based early diagnosis of dementia diseases is increasingly becoming a subject of debate against the background of the Karlsruhe ruling.

**Discussion:**

Needs-oriented and resource-enhancing approaches can make a significant contribution to improving the quality of life of people with dementia. The discussion on the issue of “assisted suicide” should include questions of the dignity and value of a life with dementia.

**Outlook:**

The integrative dementia therapy model can be complemented by a religion- and spirituality-based approach. Appropriate forms of psychotherapy should be scientifically evaluated.

## Introduction

More than five million people across Europe are affected by dementia [1–3], Albat (2020) even indicates the number as approximately 10 millions [4]. The prevalence increases with age: while it is about 5% among those over 65, the figure rises to 20% among those over 80. Alzheimer’s disease (AD) is the most common form of dementia; often there is a comorbidity with vascular dementia. At the beginning of 2020, the German Federal Constitutional Court lifted the ban on commercial support for suicide in Germany, thereby putting suicide assistance on a legalised basis.

Although in the broader field, words such as “self-determined death” and “death with dignity” have become popular, we use the term “assisted suicide” throughout the text because its equivalent “assistierter Suizid” is the term most commonly used in Germany, whereas the concept of “euthanasia” may induce associations with Nationalsocialist murders as described below.

In mid-June, four university professors presented a draft law to clearly regulate assisted suicide. In it, the legal responsibility for assisting suicide was assigned to medical decision-making. The medical ethicist Urban Wiesing says “Our proposal is appropriate for a modern, pluralist society, because it does not explicitly express any defining ethics of successful living and successful dying” [5].

In this context, questions about medical humanities in AD arise, making it necessary to take medical-historical and ethical aspects into account.

The term “medical humanities” refers - in its original meaning - to an interdisciplinary field at the interface of medicine and all academic disciplines relating to human beings, whether as individuals or collectives [6]. Greaves and Evans describe medical humanities as a second generational response to the shortcomings of a medical culture dominated by scientific, technical and managerial approaches [7]. According to Greaves and Evans, the first response has come in the 1960s and 1970s and has led to the emergence of medical sociology and of social history of medicine [7]. They describe medical humanities as “additive” in the sense of “complementing medical science and technology through the contrasting perspective of the arts and humanities, but without either side impinging on the other” or as “integrated” in the sense of refocussing “the whole of medicine in relation to an understanding of what it is to be fully human” [7]. While the authors agree with this integrated definition, in popular discourse it is no longer used in this manner, because in a sort of third generational response medical humanities now are predominately guided by bioethical perspectives (Compare: *Division of Medical Humanities and Bioethics* - University of Rochester Medical Center) - a development tending to restrict the essential holistic approach towards the sick individual. The integrated medical humanities have to be enhanced because “health is more than the absence of disease. It is also more than a biological phenomenon. It is inherently social, psychological, cultural and historical” [8]. Therefore our article itself is “an exercise in radical interdisciplinarity” [8].

## Results

### From the history of Alzheimer’s disease

In 1906 Alois Alzheimer (1864–1915) presented the case of Auguste D. in academia. Alzheimer had met Auguste Deter in Frankfurt in 1901. This patient had experienced memory detriment, persecution complex, and psychologic alterations. After her death, Alzheimer and his former clinic director Emil Sioli (1852–1922) decided the brain material to be sent to Alzheimer’s psychopathological laboratory in Munich. Auguste Deters brain’s necropsy showed a substantial shrinking around and in the brain’s nerve cells [9].

In 1910, Emil Kraepelin (1856–1926) classified dementia into senile dementia and presenile dementia. In the eighth edition of his psychiatric textbook, he called the latter “Alzheimer’s” disease for the first time [10], after Alois Alzheimer, who had discovered pathological features of presenile dementia [11] (see further [12–14]). By including the time of manifestation, Alzheimer’s disease was subclassified into early- or late-onset [15], although “psychopathologically and morphologically there is no difference [...]” [16]. 

Towards the end of the 1960s, researchers including Hans Lauter, later professor of psychiatry at the Technical University in Munich, pointed out the analogous “treatment possibilities of presenile ‘Alzheimer’s disease’ and senile dementia; only then did Alzheimer’s dementia become a major topic of clinical psychiatry and basic science” [17]. Although relevant tasks of medicine and society after the work of Alzheimer and other early researchers revived only in the 1960s [17], meanwhile AD did not completely "fell into oblivion" [17]: in the Nazi era, these patients were among those to fall victim to "euthanasia" [18–20].

### Lost time – an ethical challenge for the present and the future – with a look into the past

In the famous madeleine scene in the first volume of his “Recherche”, Marcel Proust (1871–1922) posits sensory perception as a trigger of memory. “But, when nothing subsists of an old past, [...] more persistent, more faithful, smell and taste still remain for a long time, like souls, remembering, waiting, hoping [...]” [21]. This access to the “debris” of encoded memory content via sensory perception –well-known as the "madeleine effect" or "Proust effect"– makes the loss of the sense of smell, a typical initial manifestation of Alzheimer’s disease, appear particularly serious.

The more the individual living with dementia is deprived of core functions of his or her personality– beginning with orientation– the more painfully he or she is exposed to the danger of a practical loss of dignity. Basic affective-motivational and attentional systems, including positive affect/approach, fear, frustration/anger, and effortful control [22] represent the core of the individual’s personality. Especially in the early stages of dementia, the persons concerned become aware of the deterioration of these functions which they might perceive themselves as a loss of dignity.

Honor and dignity are the essential ethical and psychological attributes of an individual as a subject of highly moral behavior and creative activities for the production of material and spiritual values, including his/her self as the supreme value (Ribalka В. Psychology of honor and dignity of a personality,Unpublished). Dignity is one of the most important dimensions of a human being, dignity is the result of the interaction between 3 different internal representations: „the Self, the others’ perception about the Self and the perception one’s Self with others in a social context “ [23]. It has been demonstrated that patients’ sense of dignity is granted if physical distress is low, treatments are noninvasive, independence, autonomy, and privacy are ensured, relationships are meaningful and care is dignified [23].

### Integrative Dementia therapy models

Art, dance and music therapy enable a healing approach to the “borderline experience” of fully recognisable human dignity ( [24], see further [25]). As part of the art-therapeutic Artecura project “Faces of people with dementia” by Claudia Büeler, for example, more than 1000 drawings and paintings by and with people suffering from dementia were created in 2003 in old people’s homes in Germany and Portugal, with the portraits acting “like a bridge” to the lost self [26]. For people suffering from dementia, who are less and less able to verbally communicate their cognitions and emotions, dance offers itself as a means of self-efficacy and connection to fellow human beings. “Lose your head and find your senses again,“ formulated the founder of Gestalt therapy, Fritz Perls (1893-1970). People who have “lost their head” due to illness find important access to their feelings in therapeutic dance [27]. In the early stages of a dementia process, creative dancing can at least temporarily let the person concerned be distanced from the anticipation of cognitive and physical decay.

In his autobiographical work of 1890, “The Story of a Child”, the French naval officer and writer Pierre Loti (1850–1923) describes his grandmother, her “deranged soul”, and her patriotically moved singing of French revolutionary anthems [28]. Music therapy can open a door to lost identity by including songs associated with personal memories [29]. Such an individual form of therapy can be provided by experienced and committed nursing staff, as well as by relatives or volunteers [29, 30]. Inclusive choir groups can also strengthen the self-esteem of people with dementia, because, “singing is fun, singing does you good, singing makes you cheerful and singing encourages. Singing makes you happy, because singing has charm, the notes hold us in their arms” [31]. The idea of being held *in* the arms emphasizes the relationship aspect as a core element of personality, which also applies when orientation, self-confidence and biographical knowledge, rationality, autonomy and life-planning have dissolved in the neurodegenerative process.

### “Genuine” vs. “pseudo” validation therapy

Validation (lat. valēre “to be strong”, “to apply”) means “strengthening”, “affirmation”, “substantiation”; the verb “validate” can also mean “to value” [32]. The validating method has “much to do with mirroring feelings”. In the film “Head Full of Honey”, [33] the validating method leads to a depathologisation of the “cognitive paradigm”: When the character with Alzheimer’s, despite having problems with pronunciation and finding words in his native German, looks through a photo album with pictures from Venice, Italian expressions of love come fluently to his lips.

In contrast to the German-American gerontologist Naomi Feil, whose validating approach emphasizes the dignity of the dementia patient [34], the psychologist Sven Lind propagates “strategies of a biographically oriented illusory world design” [35]. It is not the “being held *in* the arms” as in music therapy, but the “being taken *by* the arm” that Lind seems to favour, with such methods as simulation of reality in his “dementia villages”. However, even in advanced stages of the disease, those affected often still have a feeling for the authenticity of the person speaking to them. Three years before Sven Lind’s strategy of creating an illusory world, the medical ethicist Maartje Schermer presented a reality-based approach to people with dementia [36], in which an audio cassette [37] of relatives speaking in the calming style of a crisis intervention gives the lonely individual living with dementia the closeness of familiar people (simulated presence therapy, SPT). It must be mentioned that this description of someone living with dementia seems to reflect a personal viewpoint.

However, an up-to-date systematic review is unable to give a categorical statement on the effectiveness of SPT in treating behavioural and other psychological factors. Further valid studies are needed for this purpose [38]. The same applies to the strategies of Feil on the one hand and of Lind on the other hand.

Another way to alleviate loneliness and pain is to have robots, such as the lifelike interactive seal “Paro” [39]), read or sing texts. The practical tests conducted so far have been favourable, and the cuddly toy has been well received by patients [40]. The use of trained or untrained animals, Animal-assisted therapy (AAT) improves human health. „In patients with dementia, interaction with animals seems to have a positive influence on aggressiveness and anxiety and to ameliorate quality of life and relationship skills “ [41]. AAT targeting their specific needs and interests may be a beneficial and effective complementary treatment especially of behavioral and psychological symptoms of patients suffering from dementia [42]. Petersen et al. found out that treatment with the Paro robot decreased stress and anxiety and resulted in reductions in the use of psychoactive medications and pain medications in elderly clients with dementia [43]. Pet therapy is efficient in improving depressive symptoms and cognitive function in residents of long-term care facilities with mental illness [44]. AAT may have an apparent beneficial effect in apathy [45] (see further [46]).

### Quality of life: dimensional and standardized

Just like anyone else, people with dementia need interaction with their social environment as a key element of quality of life. Dementia researcher Martin Dichter et al. list family, social contacts, relationships, living environment, religious beliefs, security and enjoyment of activities as essential individual dimensions [47].

The nursing scientist Annette Riedel emphasises the “subjectivity of the phenomenon of pain” [48] when assessing quality of life. A study by the Clinical Department for General Anaesthesiology, Emergency and Intensive Care Medicine of the Medical University of Graz contrasts the earlier hypothesis of a generally reduced pain sensation in geriatric and/or dementia patients with more recent, differentiated research findings [49]. For a correct interpretation of the expressions of cognitively impaired persons it is important to know their individual background respective norms, values and life concepts. “The assessment is carried out systematically, at best in the context of an ethical case review” [48].

Particularly promising in this context is the world’s first “experiment in comparative ethics advice in real time”, which enables anonymous comparative analyses by ethics committees throughout Germany [25]. This standardized comparison of good clinical-ethical practice can lead to promising insights for the future whereas an individual approach, as reflected in the plurality discourse below, still remains a valid postulate.

Socially creative and psychotherapeutically validating approaches in dementia therapy should be further researched and used especially with reference to their ethical aspects. 

For the social and medical treatment of people suffering from dementia, the recent Karlsruhe judgement on assisted suicide could trigger developments that require scientific and medical-ethical discussion.

On Ash Wednesday, 26 February, 2020 the federal constitutional court of Karlsruhe has enacted a fundamental judgment: The paragraph 217 of the penal code whereupon a person who businesslike supports the suicidal intention of another person will be punished by imprisonment up to 3 years was declared as unconstitutional.

## Discussion

### “Positive biomarkerization” and assisted suicide – what next?

According to the President of the German Medical Association, Dr. Klaus Reinhard, the “bombshell” decision from Karlsruhe [50] of 26 February 2020 legalising commercial assisted suicide means that “society as a whole must find [...] ways and means to prevent organised assisted suicide from leading to a normalisation of suicide” [50]. High-ranking media reports and leading clinical European Guidelines connect – quasi inevitably – the symptoms of neurodegenerative human diseases – cognitive deficits, physical limitations, psychiatric symptoms, loss of individual and social structures – with the burden on the relatives providing care [2]. 

In view of the genetic tests available, this equation becomes particularly controversial. Before testing for genetic risk factors, the patient is informed about possible consequences of the medical findings. This is the case, for example, with the gene for apolipoprotein E, which can be analysed from blood serum and whose allelic variant E4 is expressed as the strongest genetic risk factor in more than half of AD patients [51, 52]. Standard analyses of cerebrospinal fluid (CSF) markers (beta-amyloid [1–42]/ [1–40] ratio, phospho-tau) are also considered predictive of Alzheimer’s disease [53]. The combination of a decreased CSF amyloid β42 and an increased CSF tau/phospho-tau as proclaimed criteria in the revised NIA-AA (National Institute on Aging (NIA) at National Institutes of Health and the Alzheimer’s Association) are based on the best available science now but are not yet ready for generalised use. It should be reserved to specialised centres with appropriate knowledge and facilities [54]. 

Indeed it may not appropriately measure the item of concern: the international standardised registration shows a low rate of pure AD pathology; it is the combination of vascular dementia and AD that is more often present [55]. Up to now there is no generally accepted procedure for a biomarker-validated prediction of AD in mild cognitive impairment (MCI) (DGPPN). Thus, the question arises as to how total tau, phospho-tau, beta-amyloid ratio as well as new markers, should be validated as risk factors [55]. „The extent to which knowledge per se of being affected by AD has a value will continue to be the subject of societal and political discussion “ [54] (see further [56]). To date, there is no effective prophylaxis against Alzheimer’s dementia, and none of the so-called antidementia drugs has been approved by the authorities for the treatment of mild cognitive impairment – a possible precursor of Alzheimer’s disease [57]. In this context, the will to live of even a solely “biomarkerized” risk carrier for dementia could be equated to an increasingly unacceptable burden on relatives (see [58]). The dignity and value of a life – being taken to its barbaric perversion in the darkest chapter of German history [18, 20] – with the manifest or even predicted disease would be relativised in a way that is incompatible with a humane or Christian ethical view. "All ethics has a religious dimension" [59] (see further [60]). "Christian insights concerning death, suffering, human nature and human creatureliness can help to expose more fully the moral issues at stake in some of the dilemmas faced by remote spaces" [59]. The Christian contribution to medical ethics has been huge and constructive: a core belief in the intrinsic value of human life, respect for which we as religious human beings, are accountable to God [61].

Referring to the predictive genetic diagnosis of Huntington’s disease, which also leads to dementia, Christiane Lohkamp, member of the German National Ethics Council, former chairwoman of the charity Deutsche Huntington-Hilfe e.V. and member of the International Huntington Association (IHA), emphasises: “The doubts about the gene status no longer exist, but they are replaced by other questions” [62]– namely the question of recourse to “assisted suicide”. 

The fact that – even from the medico-ethical point of view – the compass only goes in one direction, namely that of plurality, like described in the introduction, does not seem helpful. One of the tasks of medical ethics is to work out basic ideas about “successful” living and dying in our modern society. The more a society moves away from religious, pastoral and philosophical positions on the question of what is good living and dying, the more it needs concrete and differentiated guidance from the medical-ethical side. A basic problem of anticipatory provisions should also be taken into account, as the case of Walter Jens (1923–2013) notably demonstrates: The rhetoric professor had consistently advocated a self-determined death and the legalisation of “assisted suicide”, but distanced himself from his former positions by his living will after the onset of his dementia in 2004 [63]. According to Hans Förstl, the discrepancy between individual ideas about the future and the experience of the concrete situation is “hardly bridgeable”. “Human beings can generally cope with new situations” [63]. The physician, too, who is to be assigned the legal responsibility for assisting suicide, needs a medical-ethical statement on legalised “assisted suicide”, one that goes beyond the view of a plurality discourse that “it is good because it is permitted”. 

Fine arts, music and physical movement, especially dance, can be used in many ways with personal caregivers as well as psychotherapeutically to improve the quality of life of people with dementia. Validating procedures in communicating with people with dementia require high levels of empathy, patience and specialist training. The staging of “illusory worlds” can contribute to better the quality of life of gerontopsychiatric patients, but it requires great skill and critical attention from an ethical perspective. Robot-supported help against loneliness and for pain relief is well accepted by patients, according to previous findings [39]. Sensory stimulation like shiatsu and acupressure, aromatherapy, massage/touch therapy, light therapy, sensory garden and horticultural activities, snoezelen multisensory stimulation therapy, transcutaneous electrical nerve stimulation and behaviour management techniques may also be taken into account in the treatment of patients with AD [64].

### Concluding remarks

In order to find individually appropriate answers to the “other questions” mentioned above (Lohkamp, [62]), an integrative dementia therapy model with art, dance, music and validation therapy can be helpful, possibly combined with a religious/spiritual approach [64–68], especially since older patients are more likely to be willing to accept this. Although the scientific evaluation of religiosity- and spirituality-centred interventions in persons with dementia remains a task for the future, a spiritual anamnesis (e.g. [69]), should however, always be carried out as far as possible in order to use the additional information for therapy.

## Data Availability

Guaranteed.

## Abbreviations

AAT: Animal-assisted therapy
AD: Alzheimers Disease
CSF: Cerebrospinal fluid
DGPPN: Deutsche Gesellschaft für Psychiatrie und Psychotherapie, Psychosomatik und Nervenheilkunde
IHA: International Huntington Association
MCI: Mild cognitive impairment
NIA-AA: National Institute on Aging (NIA) at National Institutes of Health and the Alzheimer’s Association
SPT: Simulated presence therapy
